# Analysis of Research Directions on the Rehabilitation of Patients with Stroke and Diabetes Using Scientometric Methods

**DOI:** 10.3390/healthcare10050773

**Published:** 2022-04-21

**Authors:** Ileana Pantea, Angela Repanovici, Maria Elena Cocuz

**Affiliations:** 1 Faculty of Medicine, Transilvania University of Brasov, 500036 Brasov, Romania; ileana.pantea@unitbv.ro; 2 Faculty of Product Design and Environment, Transilvania University of Brasov, 500036 Brasov, Romania; 3 Department of Fundamental Disciplines and Clinical Prevention, Transilvania University of Brasov, 500036 Brasov, Romania; maria.cocuz@unitbv.ro

**Keywords:** diabetes, stroke, scientometrics, Web of Science

## Abstract

The multidisciplinary approach to the rehabilitation of patients with stroke and diabetes has been followed in this article by a review of the literature published in the Web of Science in the last ten years. A review of the literature was performed using scientometric methods. VOS Viewer software was used to determine the research directions in this area. Scientometric analysis has extracted relevant published scientific output that treats diabetes and stroke. Studies based on qualitative research and the conclusions of these studies were analyzed. The clusters with the keywords used in the title and abstract by the authors who published in the Web of Science were reviewed and research directions in the field were formulated. The proper care of diabetes and its numerous consequences, including stroke and its neurologic complications, necessitates the fast identification of research findings in various types of medicines and their efficacy when applied to various patient groups, such as diabetic patients, whose recovery after a stroke is similar to that of a nondiabetic patient following hemodynamic stabilization, although it takes longer and has poorer outcomes. The limitations of the study refer to the fact that the data reviewed are from the Web of Science only.

## 1. Introduction

### 1.1. Multi-Perspective Approach

The multi-perspective approach involves exploring the challenges and opportunities involved in developing rigorous and coherent research methodologies to capture complex phenomena by using multiple perspectives to explore the same phenomenon [1]. This multi-perspective approach was designed to facilitate patient-centered design and evaluation. We used diabetes and stroke scientific production to analyze field observations, structured interviews, and document analysis to collect and analyze user workflow patterns, decision support goals, and diabetes patient interaction preferences. 

The purpose of this meta-analysis using scientometric methods is to identify possible approaches to the recovery of post-stroke diabetic patients.

Although there are still ongoing studies to identify the particular approach of the diabetic patient in post-stroke recovery to date, no particular method has been identified for them compared to patients without diabetes. The only difference is the extension of the recovery period in diabetic versus non-diabetic patients.

Diabetes mellitus may influence the post-stroke clinical evolution, especially in the initial phase, increasing the extension of the cerebral injured area [2]. Few studies have been aimed at studying the influence of diabetes on functional outcomes after stroke, and their results are not conclusive [3].

Diabetes is a chronic disease with a growing prevalence that affected a population of approximately 415 million in 2015, and will reach approximately 642 million in 2040 [3]. Diabetes increases the risk of stroke four-fold.

Analyzing the literature shows that there are various implications that diabetes has on different patients. We present some situations that the authors in the field presented in their published articles:

- For stroke, diabetes is an independent risk factor and is associated with increased mortality and morbidity [4].
- In diabetic patients, there is a high proportion of ischemic strokes versus hemorrhagic strokes, and heart attack is the most common type of stroke in these patients. This fact is due to the multiple microvascular damages and the simultaneous presence of arterial hypertension in diabetic patients [5,6].
- Diabetic patients have a greater functional disability, long-term hospitalization, and also a higher risk of dementia [7,8].
- Multicenter studies have shown that approximately 20–33% of stroke patients have diabetes [9,10,11].
- However, some studies suggest that diabetes has no influence on motor and functional outcomes within the acute and post-acute phase after stroke. Further research should be conducted to investigate motor recovery in a longer-term period and with larger samples.

In conclusion, the multidisciplinary approach that involves the diabetologist and neurologist in the case of a diabetic patient with a stroke is the key to success for a complete, functional, and integrated rehabilitation.

### 1.2. Scientometric Methods

Scientometric methods involve the quantitative analysis of the generation, dissemination, and use of scientific information, and allow the determination of large and emerging trends in scientific research in a particular field of research, based on statistical analysis of databases and the use of qualitative filters (topics, keywords, magazines). At the same time, it allows the review of the development of research over time or the geographical and organizational distribution of scientific production. The primary scientific data used for scientometric research are the authors, their papers, bibliographic references, and quotations received [12].

Scientometric examinations allow the identification of the most current research topics in a certain field, and the identification of the most quoted papers and authors who have addressed a specific topic. It is possible to determine which countries, institutions, and journals have the greatest influence on the development of science in a particular field and to analyze how the interest in a particular scientific discovery varies over time [13,14].

“A fast-growing trend is the increase of systematic reviews conducted with the assistance of science mapping tools” [13]. A science mapping tool typically takes a set of bibliographic records of a research field and generates an overview of the underlying knowledge domain, very quickly. “A scientometric overview of a field of research provides a valuable source of input to conducting systematic reviews, especially in situations when relevant and up-to-date systematic reviews may not be readily available or accessible” [14].

## 2. Materials and Methods

The Web of Science database was chosen as a representative sample of the scientific population in the field of diabetes and stroke. Using bibliographic data from this database, this paper seeks to identify current and future directions of research in the field of “diabetes” AND “stroke recovery”. In this respect, the search in the database was limited for the time period 2011–2021, the last 10 years. The works were searched by topic, respectively ((“diabetes” OR “diabetes mellitus” OR “type 2 diabetes” OR “type 1 diabetes”) AND “stroke recovery”). The search was performed on 28 May 2021, and the resulting works were refined according to the type of documents, and only the scientific articles, in English and in Open Access, were selected, resulting in 46 papers.

Primary data were downloaded as plain text files from the Web of Science (WoS) database. The results were examined using VOS Viewer software version 1.6.16 [15], which allows scientific mapping to analyze the content of titles and abstracts of scientific publications. Thus, the VOS Viewer term identification function was used to systematically identify key terms in the database (co-word analysis) and organize large amounts of text in a semantic map, ignoring the elements related to the structure of abstracts and copyright statements that might be included.

To prepare the terms for mapping purposes, VOS Viewer measures the link between the terms using the power measurement and suggests how many terms should be included in the map.

In this case, of the 1663 terms identified, 90 were used more than 2 times. The groups were analyzed, and the research directions were identified. In addition to this analysis, collaboration and quotation networks have been identified, details of which will be provided in the next section.

The scientometric study includes seven steps, according to Table 1, starting with the formulation of the problem, the establishment of protocols and research criteria, and the extraction of data, which were subsequently analyzed, synthesized, and discussed.

Analysis and interpretation of scientometric research data.

**Figure 1:**
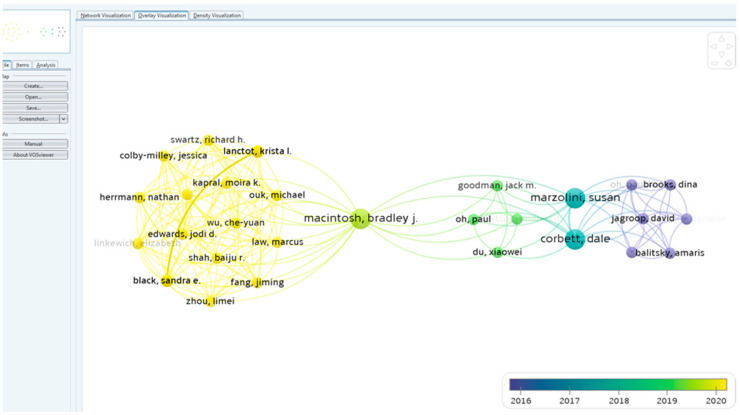
Authors, visual map according to number of publications.

**Figure 2:**
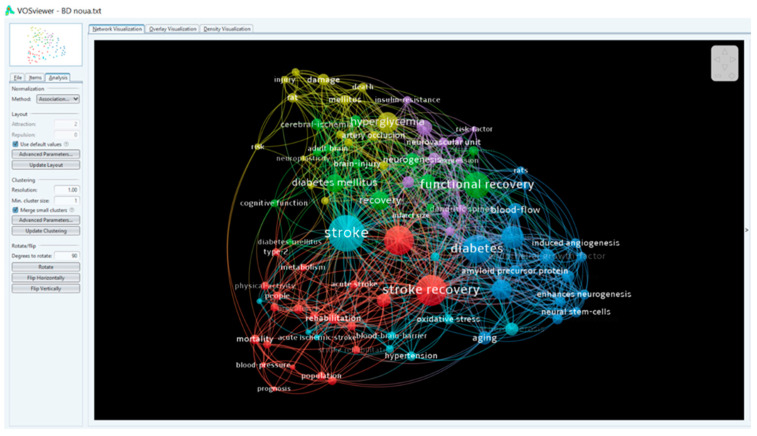
Keyword density map.

**Figure 3:**
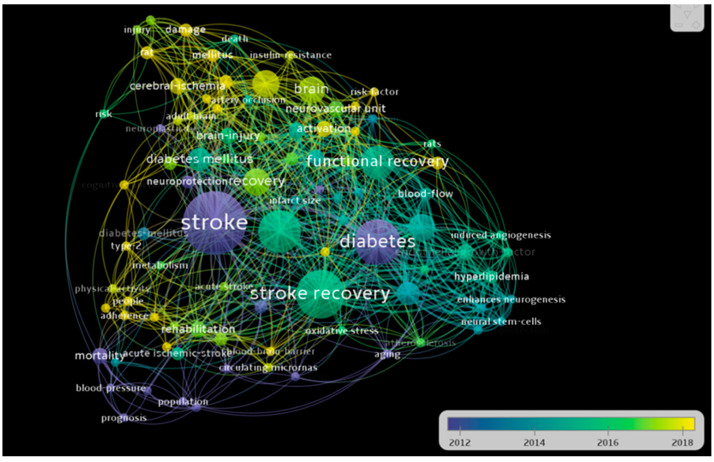
Keyword density map.

**Figure 4:**
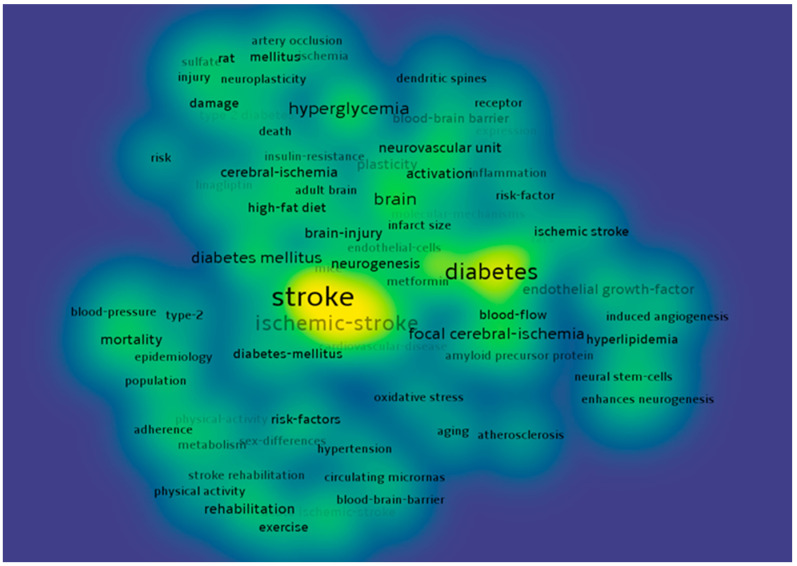
Keyword density map.

A total of 214 authors contributed to the writing of the 46 papers in the 10 years analyzed. The main authors are presented in Figure 1 according to the number of publications. There were 90 terms that appeared at least twice, and they are distributed in 6 clusters, according to Figure 2, Figure 3 and Figure 4.

**Figure 5:**
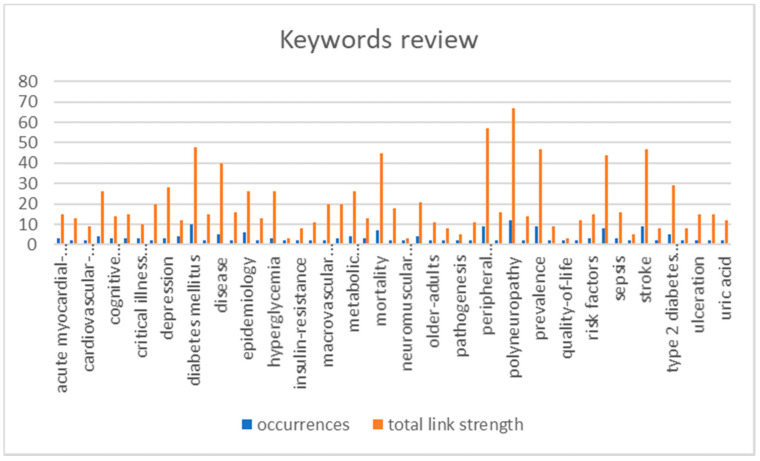
Keywords’ occurrence. The words that have the highest rate of occurrence are: neuropathy, disease, and type 2 diabetes mellitus. Epidemiology, diabetes mellitus, mortality, risk factors, peripheral neuropathy, prevalence, stroke, polyneuropathy.

Using the keyword list and Excel, the keyword figure was obtained (Figure 5).

## 3. Results

Following the review of the 46 papers, the authors identified 21 studies and articles reviewing the specific literature (Table 2). The articles were reviews, randomized studies, and meta-analyses. Some articles indicate the number of participants, while others do not, indicated as n for not specified in Table 2.

The authors have also reviewed the articles delimiting the research directions in the six clusters presented in Figure 2, Figure 3 and Figure 4. Table 3 summarizes the results.

## 4. Discussion

This article presented a new method of literature review, using scientometric methods. The method can be replicated by PhD students and researchers, in order to quickly obtain an image of any field researched. Studies in human models have shown that the therapeutic balance of diabetes is a very important factor in the rehabilitation of diabetic patients after stroke. The review of the articles and research directions led to conclusions that are important in the analysis of solutions for patients with diabetes and stroke. Articles studied in either animal or human models have highlighted several research directions for post-stroke rehabilitation programs.

The investigation of the articles led to different conclusions, which we present below in six directions of research.

1. Identification and possible modification of aggravating risk factors for stroke patients

Obesity associated with diabetes are factors that worsen the prognosis of recovery after stroke.

Ischemic strokes occur predominantly in diabetic patients with arrhythmias and acute coronary syndromes, while accidental hemorrhagic strokes occur predominantly in smokers and alcoholics [31].

2. The influence of mental disorders on post-stroke recovery

Depression increases the risk of readmission to recovery centers and may contribute to dementia. A diabetic patient-centered recovery strategy alleviates the risk of depression and multiple post-stroke metabolic complications [16].

3. Understanding post-stroke vascular remodeling processes

In young people, remodeling processes are active and long-lasting compared to the elderly, in whom remodeling is slowed down due to the presence of other comorbidities, such as diabetes, atherosclerosis, and dyslipidemia.

Glycemic variations in diabetics have negative effects on angiogenesis, so stimulating it in stroke patients is not currently an optimal solution for neurological recovery [29,54].

4. The role of physical activity on post-stroke recovery

Information on this issue is still controversial. The indications for physical exercise in the early post-stroke phases showed progress in recovery, but neither their intensity nor their optimal duration was established. The lack of data in the literature indicates that early post-stroke mobilization is limited [32].

5. The effects of drug therapies and dietary supplements

The effects of hypoglycemic medication on the risk of stroke are heterogeneous.

Sulfonylureas and metformin appear to have a potential protective effect in diabetic patients with hemorrhagic stroke [29].

Studies of DPP4 inhibitors in diabetic patients have shown a reduced risk of stroke. Complementary studies are needed to show possible effects of reducing brain damage in the case of stroke [19].

The use of long-term dietary supplements enriched with potassium and magnesium seems to be beneficial for post-stroke recovery, but the results require further studies to substantiate the indication [25].

The administration of micro-RNA biomarkers (miRNAs) in stroke therapy could be an alternative to long-term sequelae, but studies are still ongoing [21].

Another class of innovative antidiabetic drugs that inhibit SGLT2 with complex mechanisms of action do not increase the incidence of stroke. SGLT2 inhibitors produced a 50% decrease in hemorrhagic stroke compared to a placebo [55].

6. There are a number of factors that can cause patients to drop out of rehabilitation programs. These are related to transportation problems to the recovery centers, long distances to reach these centers, boring exercises, lack of a companion, and limited time.

Therefore, future studies should focus on ways to overcome these barriers to encourage the participation of these patients in rehabilitation programs.

## 5. Conclusions

Following the scientometric research and the analysis of the specialized literature, the authors wish to raise the awareness of the specialists regarding the following aspects.

Proper management of diabetes and its many complications, including stroke neurology, requires the rapid identification of research results in different types of therapies and their effectiveness applied to various categories of patients.

The recovery of the diabetic patient after stroke after hemodynamic stabilization is the same as in the case of nondiabetic patients, but involves a longer period of time and has poorer results.

SGLT2 inhibitors had no effect on overall cerebrovascular events; however, results for stroke after using them vary depending on the kind of stroke, with a potential benefit for hemorrhagic stroke prevention. Further prospective trials comparing the effects of SGLT2 inhibitors on different stroke subtypes are needed.

Finally, we conclude that scientometric methods allow a rapid and efficient analysis of the research directions generated by scientific production in the field, making a decisive contribution to improving the approach to the disease and long-term treatments of patients with diabetes and stroke.

## 6. Limits of the Study

The scientometric analysis must be interpreted taking into account the limitations of the research. First, the results are limited to publications (articles and papers presented at conferences) published in 2011–2021 and indexed in the Web of Science database.

However, this scientometric analysis has allowed us to identify the main actors and research directions in the field in recent years. The results of the research show that many of the previous interests are still relevant today.

## Figures and Tables

**Table 1:** Scientometric study stages.

No.	Steps	Description
1	Formulation of the problem	Mapping, bibliometric analysis of publications using descriptors and identification of research directions.
2	Research criteria	Subject: ((“diabetes” OR “diabetes mellitus” OR “type 2 diabetes” OR “type 1 diabetes”) AND “stroke recovery”)
3	Database used for research	Claryvate analytics, Web of Science (WoS) Accessed on 28 May 2021
4	Eligibility criteria	Filter 1: years of publication (2011–2020) Result: 1663 documents Filter 2: articles Filter 3: English Filter 4: Open Access Result: 43 documents
5	Data extraction	Bilingual format
6	Analysis and synthesis of results	Qualitative (descriptive) and quantitative (bibliometric) using VOS Viewer
7	Discussions	Analysis of the data gained

**Table 2:** Studies and reviews, literature articles. n = not specified.

Source	Number of Participants	Trial Type	Duration of the Trial	Title of the Study
[16]	23,579	Randomized trial	2003–2013	Depression and Diabetes Mellitus Multimorbidity is Associated with Loss of Independence and Dementia Post-Stroke
[17]	46	Review	n	Aerobic Training and Mobilization Early Post-Stroke: Cautions and Consideration
[18]	n	Review	n	Effects of Angiotensin-II on Brain Endothelial Cell Permeability via PPAR-alpha Regulation of Para- and Trans-Cellular Pathways
[19]	n	Review	2011–2019	Dipeptidyl Peptidase-4 Inhibitors for the Potential Treatment of Brain Disorders: A Mini-Review with Special Focus on Linagliptin and Stroke
[20]	70	Randomized trial	14 days	Sleep and Cognitive Function in Chronic Stroke: A Comparative Cross-Sectional Study
[21]	58,265	Meta-analysis	n	Cerebral Vascular Injury in Diabetic Ischemia and Reperfusion
[22]	n	Review	n	Occupational Physical Activity in Young Adults and Stroke: Was It Due to My Job?
[21]	n	Article	n	Impact of microRNAs on Ischemic Stroke: From Pre- to Post-Disease
[23]	160	Trial	April–June 2014	Increased Expression of STIM1/Orai1 in Platelets of Stroke Patients Predictive of Poor Outcomes
[24]	n	Article	n	Stroke in Women: Risk Factors and Clinical Biomarkers
[25]	291	Study	2009–2013	Intake of Potassium- and Magnesium-Enriched Salt Improves Functional Outcome after Stroke: A Randomized, Multicenter, Double-Blind Controlled Trial
[26]	374	Study	January 2005–May 2010	Clinical and Imaging Correlates of Outcome after Intracerebral Hemorrhage
[27]	78	Clinical trial	12 weeks	Rationale and Design to Assess the Efficacy and Safety of HT047 in Patients with Acute Ischemic Stroke: A Multicenter, Randomized, Double-Blind, Placebo-Controlled, Parallel-Group, Phase II Trial
[28]	489	Trial	2004–2009	A New Prognostic Scale for the Early Prediction of Ischemic Stroke Recovery Mainly Based on Traditional Chinese Medicine Symptoms and NIHSS Score: A Retrospective Cohort Study
[29]	n	Review	n	Cerebral Neovascularization in Diabetes: Implications for Stroke Recovery and Beyond
[30]	10	Study	n	Resistive Training Improves Insulin Sensitivity after Stroke

**Table 3:** Diabetes and stroke research directions, determined by the six clusters.

Cluster	Conclusions	Supporting Document
1	The post-stroke motor function is improved by physical activity due to low levels of epinephrine and norepinephrine.	[22]
	Resistive training can decrease post-stroke glucose metabolism and thus increase post-stroke survival.	[29]
	Age is an important factor in post-stroke recovery: patients under the age of 56 are more likely to recover than those over the age of 56.	[27]
	The combination of diabetes mellitus (DM) and high blood pressure (HBP) entails a poorer recovery in the first 3 days after the stroke.	[27]
	Patients with diabetes and heart disease are more likely to have a predominantly ischemic stroke, while smokers and alcoholics are more likely to have a hemorrhagic stroke.	[31]
	Mobilization of post-stroke patients in the first 6 h exacerbates injuries, whereas after 24 h from stroke the mobilization has beneficial effects.	[32]
	Late recovery in special centers encounters difficulties in transporting patients to these centers and thus the sequelae can no longer recover	[32]
	DM decreases the effectiveness of thrombolysis and increases the risk of post-trobotic hemorrhage.	[33]
	DM does not affect motor and functional recovery in the acute and post-acute phase of stroke.	[34]
2	DM impairs cortical plasticity.	[35]
	DM affects post-stroke neovascularization, thus preventing post-stroke recovery.	[36]
	Diabetes impairs spatial memory and hippocampal neurogenesis in ischemic stroke.	[37]
	Diabetes increases the risk of dementia by 85% compared to non-diabetic people.	[16]
	The association of dementia with diabetes in stroke patients leads to poorer results in post-stroke recovery.	[38]
	The recovery rate was slower in patients with stroke and diabetes. DM exacerbated anxiety and cognitive decline.	[39]
	DM highly increases neurovascular damage and thus depreciates post-stroke recovery.	[37]
	Obesity with diabetes determines reduced neurogenesis and impaired neuroplasticity after stroke.	[40]
	Obesity induces a reduced post-stroke recovery.	[41]
	Atrial fibrillation appears to affect post-stroke recovery.	[42]
	Gender-related: Women appear to be more likely to have a stroke than men.	[43]
	In the first 3 months after the stroke, mortality is higher in the event of hemorrhagic stroke.	[31]
	Intake of N acetyl seryl aspartyl lysyl proline (AcSDKP) has led to improved neurological functional recovery in rats with diabetes.	[23]
	Thiazolidinedione treatment in diabetic stroke patients has intensified post-stroke functional recovery by decreasing infarct volume and vasodilation.	[44]
	Long-term administration of potassium and magnesium benefits post-stroke recovery.	[25]
	Sulfonylureas and metformin used in hemorrhagic stroke causes angiogenesis and has a high safety profile.	[37]
	Metformin mediates post-stroke recovery by increasing angiogenesis.	[29]
3	In the elderly, 40% have moderate functional post-stroke impairment, but people over 85 show slower rehabilitation.	[45]
	Angiogenesis in diabetic patients is greatly slowed down.	[46]
	Glycemic control prevents the decline of neovascularization and post-stroke recovery.	[29]
	Type 1 diabetes has a 4–6 times higher incidence of ischemic stroke occurrence.	[47]
	Post-stroke blood–brain barrier dysfunction (BHE) plays an important role in limiting functional recovery in diabetic patients.	[41]
	Angiotensin-II is a significant factor in increasing endothelial permeability in the brain and contributes to angiogenesis and neurogenesis.	[18]
4	Effects of DM treatment on post-stroke recovery: Antidiabetic medication such as DPP4 inhibitors, sulfonylurea. Glimepiride causes faster post-stroke recovery in obese diabetic patients.	[48,49]
	DPP4 does not decrease the risk of stroke but causes early recovery and rehabilitation in the first 3 days after a stroke.	[19]
	Obesity and diabetes worsen post-stroke recovery, and these effects are counteracted by the administration of DPP4/sulfonylurea at 3 days post-stroke, leading to early recovery.	[19,48]
	Stroke shows an increased number of neurons 6 weeks after the stroke. Diabetes causes neuroplasticity and thus this effect of increasing the number of neurons is abolished.	[48]
5	Innovative therapies in animal studies. Administration of C21 to a type 2 angiotensin-II receptor agonist on day 3 after a stroke resulted in a reduction in neuroinflammation in male animals with diabetes.	[50]
	Inhibition of TOLL-4 (TLR4) receptors in microvascular endothelial cells would reduce inflammation and improve post-stroke recovery in diabetics.	[46]
	miRNA assay would be a biomarker for the diagnosis of stroke and the evaluation of the effectiveness of stroke treatment.	[51]
6	White matter lesions in the brain occur in diabetic patients long before stroke occurrence.	[23]
	Patients with chronic kidney disease (CKD) more frequently suffer ischemic stroke than hemorrhagic stroke.	[52]
	In patients with BCR, uremic toxins cross the BHE and are thus involved in cognitive dysfunction and neurodegeneration.	[52]
	In stroke patients, the combination of CKD worsens recovery and limits the choice of therapies for stroke treatment.	[53,54]
